# Surface Oxygen Deficiency Enabled Spontaneous Antiprotein Fouling in WO_3_ Nanosheets for Biosensing in Biological Fluids

**DOI:** 10.1021/acs.analchem.3c04414

**Published:** 2024-01-04

**Authors:** Guozhen He, Tao Dong, Zhaochu Yang, Bjo̷rn Torger Stokke, Zhuangde Jiang

**Affiliations:** † Chongqing Key Laboratory of Micro-Nano Systems and Smart Transduction, Chongqing Key Laboratory of Colleges and Universities on Micro-Nano Systems Technology and Smart Transducing, Collaborative Innovation Center on Micro-Nano Transduction and Intelligent Eco-Internet of Things, Chongqing Academician and Expert Workstation, Chongqing Technology and Business University, Nan’an District, Chongqing 400067, China; ‡ Department of Microsystems (IMS), Faculty of Technology, Natural Sciences and Maritime Sciences, University of South-Eastern Norway, Postboks 235, Kongsberg 3603, Norway; § Biophysics and Medical Technology, Department of Physics, Norwegian University of Science and Technology, Trondheim NO-7491, Norway; ∥ Sensovann AS, Raveien 215, Borre 3184, Norway; ⊥ Xi’an Jiaotong University, Xi’an 710049, China

## Abstract

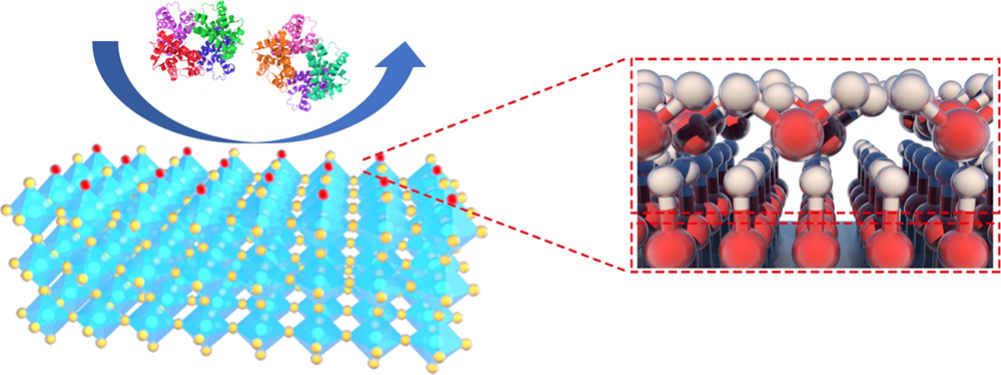

Biofouling deteriorates the performance of sensors operated in biofluids. Protein adsorption is believed to be the first step of biofouling, which also reduces biocompatibility by further inducing cell adhesion, platelet activation, and even inflammation. Current studies of antifouling coatings are focused on polymers and hydrogels, which have succeeded in remaining resistant to protein adsorption, but their application on sensor electrodes is limited due to low conductivity and biocompatibility. Here, we report a spontaneous antibiofouling strategy for sensor electrodes by controlling oxygen vacancies in WO_3_ nanosheets. Irreversible adsorption of proteins was reduced by 76% in unprocessed human plasma when electrodes were coated with WO_3_ rich in surface oxygen vacancy. These electrodes maintained 91% of the initial current density after 1 month of incubation in human plasma.

## Introduction

Biofouling is a spontaneous process characterized by nonspecific adsorption of proteins and adhesion of cells to liquid–solid interfaces.^1−4^ It will not only hinder specific recognition at surfaces of biosensors but also block electron transfer between redox species and electrodes, deteriorating the performance of biosensors.^1,5,6^ Protein adsorption is believed to be the beginning of biofouling,^7^ as the initially adsorbed layer of protein can mediate cell adhesion and thrombus formation.^4,8,9^ Protein adsorption can also trigger activation of Factor XII and adhesion of leukocytes, which further results in thrombus or inflammation.^3^ Thus, reducing nonspecific protein adsorption is critical to biosensors that operate in biological fluids.^10^ Sample treatment is a common strategy for in vitro detection, which includes dilution, centrifugation, and additions of surfactants,^1^ anticoagulants,^11^ and antiplatelet agents.^12^ Sample treatment adds extra manual operations, and it is not suitable for continuous detection in biological fluids. Alternatively, surface modification has been studied for both in vitro or in vivo sensing.^13^ The most classic approach is electrode blocking by serum albumin, whey protein, and casein,^14^ which reduces nonspecific adsorption of other proteins by the steric hindrance effect. Researchers have further developed surface functionalization by hydrogels based on poly(ethylene glycol) (PEG),^10^ zwitterions including carboxybetaine (CB), sulfobetaine (SB),^13,15,16^ their derivatives,^17^ and composites.^18^ In addition to polymeric hydrogels, Jiang et al. recently reported a superamphiphilic antibiofouling coating based on TiO_2_/SiO_2_ to microchannels.^19^ Yang and co-workers reported a Cu-doped TiO_2_ coating on the vascular stent.^20^ TiO_2_-based coating reduces nonspecific protein adsorption due to superhydrophilicity induced by ultraviolet irradiation.

Despite fruitful achievements, current antifouling coatings based on albumin and PEG hydrogels passivate the electrode surface and hinder electron transfer.^13,21^ To overcome electrode passivation, Ingber et al. recently developed a composite coating of bovine serum albumin (BSA) and gold nanowire,^22^ which successfully maintained 93% of the initial current density on the electrode after exposure to serum for 1 month.^22^ Nevertheless, immobilized BSA can unfold due to the Vroman effect^23^ or denaturation.^24^ BSA interacts with the GPIIb/IIIa receptor,^25^ promoting adhesion and activation of platelets in the blood.^26^ PEG generates multiple side effects^27,28^ and leads to the development of PEG antibodies.^29,30^ Some zwitterions are still subjected to fouling when operating in biological fluids for extended periods.^31^ TiO_2_ and its composite coating rely on ultraviolet irradiation to generate superhydrophilicity and achieve antifouling.^32^ TiO_2_ also possesses high cellular toxicity and can cause DNA damage after uptake by cells.^33,34^

**Scheme 1:**
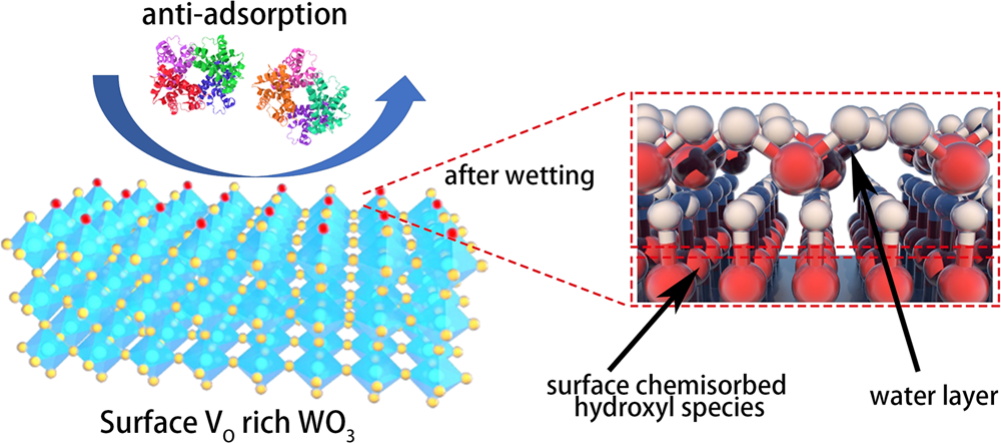
Demonstration of Proposed Antifouling Mechanisms Spontaneously Enabled by Surface Oxygen Vacancies

All in all, it still remains a major challenge to achieve a long-term antifouling coating on the electrode surface which maintains high resistance to nonspecific protein adsorption, high conductivity, and biocompatibility. In this work, we present a spontaneous antibiofouling coating based on WO_3_ nanosheets with engineered oxygen vacancies (Scheme 1). We demonstrated that WO_3_ coating rich in surface oxygen vacancy (*V*_O_) reduced protein adsorption from human plasma by 76% compared to bare ITO electrodes. After exposure to human serum and plasma for 1 month, electrodes coated with *V*_O_-rich WO_3_ nanosheets retained their current densities for more than 91%. When the concentration was as low as 500 ng/mL in the medium, the WO_3_ coating rich in *V*_O_ also exhibited low toxicity to human umbilical vein endothelial cells (HUVECs), indicating potentials in in vivo biosensing.

## Methods

### Materials and Cell Lines

Tungsten hexachloride (WCl_6_, 99.9% trace metals basis) was purchased from Sigma-Aldrich and stored in a vacuum. Absolute ethanol, oxalic acid (99.99% metals basis), and phosphate-buffered saline (PBS) were purchased from Aladdin Chemistry Co., Ltd. Isopropanol (IPA) was purchased from Macklin Biochemical Co., Ltd. Trypsin was purchased from EcoTop Bio. Dulbecco’s modified Eagle’s medium/Nutrient Mixture F12 (DMEM/F12) was purchased from Gibco. Fetal bovine serum (FBS) was purchased from Yeasen Biotechnology Co., Ltd. CellTiter-Glo (CTG) luminescent cell viability assay was purchased from Promega. Endothelial cell growth medium was purchased from PromoCell. HUVECs were purchased from Guangzhou Xinyuan Technology Co, Ltd. Human serum albumin (HSA, Cat No. A8230) was obtained from Solarbio, Beijing, China. Fibrinogen (FIB, Cat No. F3879) was obtained from Sigma-Aldrich. Lysozyme (LZM, Cat No. 9001–63–2) was obtained from Saitong, Beijing. Immunoglobulin G (IgG) was obtained from Bioss, Beijing. Commercial BCA test kits (Cat No. 3402) were purchased from Saint-Bio, Shanghai.

### Solvothermal Synthesis and Calcination of WO_3_ with Various Oxygen Defects

Detailed description of the procedure and development can be found in our previous work.^35^ In a typical procedure of solvothermal synthesis, we first added 0.2 g of WCl_6_ and 2.0 g of oxalic acid to 40 mL of absolute ethanol. After stirring for 30 min at room temperature, the mixture was transferred into a 100 mL Teflon-lined autoclave which was then sealed and maintained at 100 °C for 24 h. The product was naturally cooled to room temperature inside the autoclave and subsequently collected by centrifugation in deionized water and absolute ethanol. Collected powder, which was the precursor to WO_3_, was further calcined in different atmospheres including air, N_2_, and 20% H_2_/Ar at 300 °C for 1 h at a heating rate of 10 °C/min.

### Fabrication of WO_3_ Electrodes by Spin Coating

After calcination, WO_3_ nanosheets were dispersed into isopropanol to reach a concentration of 50 mg/mL. The mixtures were kept in a water bath and probe sonicated at 20 °C, 400 W with a pulse of 1 s on and 2 s off for various durations (8, 12, and 16 h). Sonicated mixtures were filtered with 0.45 μm syringe filters to obtain WO_3_ inks. Before spin coating, 1 × 1 cm^2^ indium tin oxide (ITO) glasses were cleaned by bath sonication sequentially in water, isopropyl alcohol, and ethanol, for 15 min each sonication. ITO glasses were further treated by a UV Ozone cleaner. As-prepared 90 μL/cm^2^ of WO_3_ inks was spin coated onto ITO glasses at a spinning speed of 5000 rpm for 30 s by a dynamic dispense technique. The spin-coated WO_3_ thin films were subsequently dried on a hot plate at 65 °C.

### Characterization of Oxygen Vacancies in WO_3_

X-ray diffraction (XRD) patterns from WO_3_ powders were recorded by using an X-ray diffractometer with Cu Kα radiation (λ = 1.5418 Å). XPS spectra were collected using monochromatic Al–Kα X-ray as the excitation source. Room temperature electron spin resonance (ESR) spectra were collected using an ESR spectrometer (298 K, 9.062 GHz).

### Characterization of Wetting Properties

The contact angles were determined with an OCA20 contact angle system. For each sample, we added 2 μL of water droplets at different locations of ITO substrates and ITO coated with WO_3_. Each sample was measure repeated four times.

### Analysis of Antifouling Using Protein Solutions

Antifouling properties of WO_3_ thin films and bare ITO substrates were conducted by incubatiing samples with solutions of selected proteins as the models. The 1 × 1 cm^2^ ITO glass samples were introduced in the wells of 24-well plates with the ITO surface facing upward. Each well was subsequently loaded with 400 μL of PBS buffer, and the glass plate was pressed to eliminate bubbles, followed by sealing plate film and allow equilibration at room temperature overnight. Following equilibration of the surface samples with PBS, the PBS was sucked off and replaced with 200 μL protein solution of either 1 mg/mL HSA, Fib, Lym or IgG dissolved in PBS. The well plate was covered with sealing plate membrane and incubated at 37 °C. After 5 min or 2 h, the glass slides were taken out, placed in a new 24-well plate, washed with 500 μL PBS buffer three times, and then washed with 500 μL deionizing water three times. After washing, the samples were dried in a drying oven with air blowing at 60 °C.

### Enzyme-Linked Immunosorbent Assay To Measure Irreversible Protein Adsorption

Enzyme-linked immunosorbent assay (ELISA) was performed by an indirect method. Sample preparation: the dried glass slides were put into a 10 mL round-bottom tube, and 2 mL of coated buffer was added. Ultrasound was performed for 20 min to elute the proteins, and the solution in the tube was collected for samples. Coating method: dilute 10× coating buffer (Cat No. RC20936; LANSO, Zhejiang, China) with deionized water to 1× working concentration. Four proteins were diluted to 1 mg/mL with coated buffer, and then a 1/2 gradient dilution was used to prepare the standard curve. We then took 50 μL of standard curve solution, HSA, IgG, Fib, and LZM samples and add them to the newly opened plate, with one hole as blank control. Followingly, all samples were sealed and incubated overnight at 4 °C. Coated plates were washed with PBS buffer solution 200 μL per well three times and added with 5% BSA blocking solution 200 μL per well. The plates were further incubated for 2 h at 37 °C and then were washed with PBST (PBS with 0.5% Tween-20) buffer solution three times. Primary and secondary antibodies (Cat No. A1363; Cat No. A1453; Cat No. A0641; Cat No. A19711; Cat No. AS014; Abclonal, Wuhan, China) were formulated using antibody diluent prior to use. In a typical experiment, we added 100 μL of a primary antibody solution into each well. After incubating at 37 °C for 1 h, we washed each sample with PBST buffer five times. We then added 100 μL of a secondary antibody solution into each well. After incubating at 37 °C for 45 min, we added 100 μL of 3′,3′,5′,5′ tetramethylbenzidine (TMB) substrate (Cat No. ab171523; Abcam) per well for color rendering. Until we observed discoloration at room temperature, we added a 0.5 M sulfuric acid solution to stop the reaction. Absorbance was measured at 450 and 630 nm with a microplate reader.

### Analysis of Antifouling Using Plasma

We incubated samples with commercial human plasma. In a typical process, we precleaned ITO substrates and as-prepared WO_3_ thin films on ITO (noted as WO_3_/ITO) with DI water and then incubated different samples in PBS at 37 °C overnight. We then carefully removed PBS with a pipet and added 300 μL of unprocessed human plasma to cover the surface of ITO sample and WO_3_/ITO samples. Let samples incubated in plasma for 5 min, 2 h, 1 day, 1 week, and 1 month. Both commercial ELISA kits and BCA test kits were used to measure irreversible adsorption of proteins for comparison. Protein standard solutions were diluted to an appropriate concentration gradient and 100 μL of solutions from every concentration were added to a 96-well plate. 100 μL of the as-prepared working solution was added to each well and incubated at 60 °C for 60 min. Absorbance was measured at 450 and 630 nm with a microplate reader.

### Electrochemical Detection of Dopamine in Human Plasma

Dopamine solutions in plasma of various concentrations were prepared by a standard addition method. Electrochemical detection of dopamine was demonstrated by square wave voltammetry (SWV), and the scan rate was set to 50 mV/s. Without biofouling on the electrode surface, dopamine was oxidated into *o*-dopaminoquinone at the surface of WO_3_ nanosheet layer, producing detectable faradaic current.

### Analysis of Cellular toxicity

We first cultured HUVECs in a medium of DMEM/F12 with 20% FBS added at 37 °C in an atmosphere of 5% CO_2_ for 24 h. WO_3_ nanosheets both rich in oxygen vacancy and without oxygen vacancy were sterilized and dispersed into the medium to reach concentrations of 0.5 to 800 μg/mL. We then incubated HUVECs with WO_3_ dispersions for 24 and 48 h. Each group was repeated three times. Cytotoxicity of WO_3_ nanosheets was examined by measuring the cell viability of incubated HUVECs using Cell Counting Kit-8 (CCK-8) and CTG Luminescent Cell Viability Assay.

## Results and Discussion

### Solvothermal Synthesis of WO_3_ with Various Oxygen Vacancies

**Figure 1:**
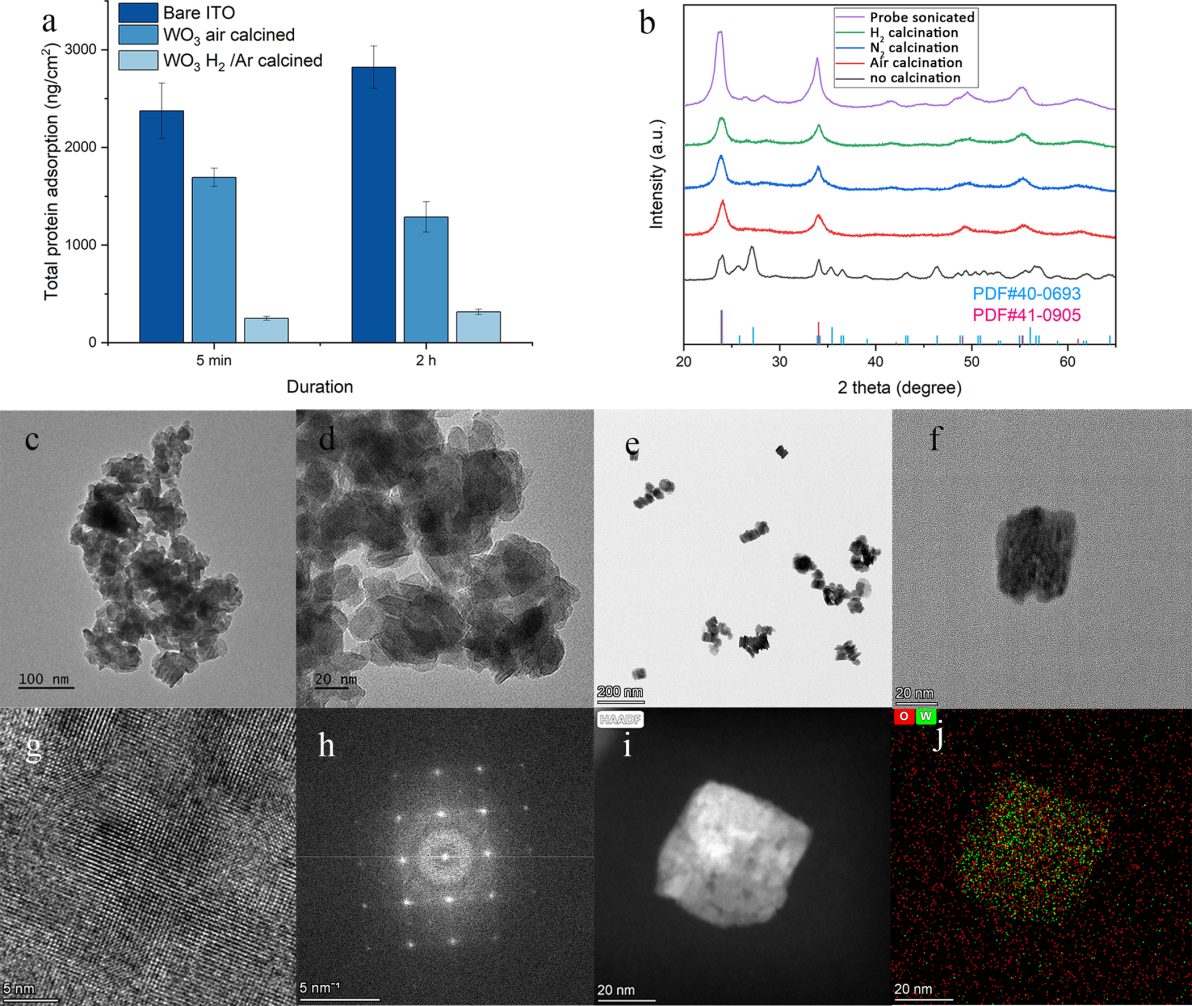
(a) Total protein adsorption from plasma to WO_3_ thin films, in which H_2_/Ar-calcined WO_3_ nanosheets were rich in surface oxygen vacancies and exhibited the best antiadsorption properties to proteins in human serum. (b) XRD patterns of the solvothermal synthesized tungsten oxide precursor, and the precursor after calcination by air, N_2_, and 20% H_2_/Ar. The air-calcined sample was also processed by probe ultrasonication for 12 h. TEM images of (c, d) solvothermal synthesized precursor and (e, f) WO_3_ nanosheets after air-calcination and probe sonication. (g, h) HRTEM and FFT and (i, j) elemental mapping of the air-calcined cubic WO_3_.

In this study, we synthesized WO_3_ nanosheets based on an optimized solvothermal method incorporated with probe sonication.^35^ XRD patterns (Figure 1b) of the synthesized product corresponded to monoclinic hydrogen tungsten oxide (H_0.12_WO_3_·2H_2_O, PDF#40–0693). As-synthesized hydrogen tungsten oxide hydrate powder contained agglomerates of nanoparticles around 20 nm diameters. The largest agglomerates were around 200 nm (Figure S1). Solvothermal products then underwent calcination in air, 20% H_2_/Ar, and N_2_, respectively. The XRD patterns of calcined products corresponded to cubic tungsten oxide (WO_3_, PDF#41–0905), which was also confined by the Raman spectrum (Figure S2). Probe sonication was applied to all calcined products in an attempt to reduce agglomerates and improve size homogeneity. Crystal structures of calcined products were observed unchanged after 12 h of probe sonication. All of the agglomerates were dispersed into free-standing nanoparticles with a size of 20 nm following probe sonication in isopropanol. As shown by transmission electron microscopy (TEM) images, hydrogen tungsten oxide hydrate of 400 nm consisted of 20 nm nanosheets (Figure 1c,d). Probe sonication in IPA broke the large clusters into smaller ones (Figure 1e,f) while preserving the structure and shape of individual nanosheets (Figure 1g–j).

**Figure 2:**
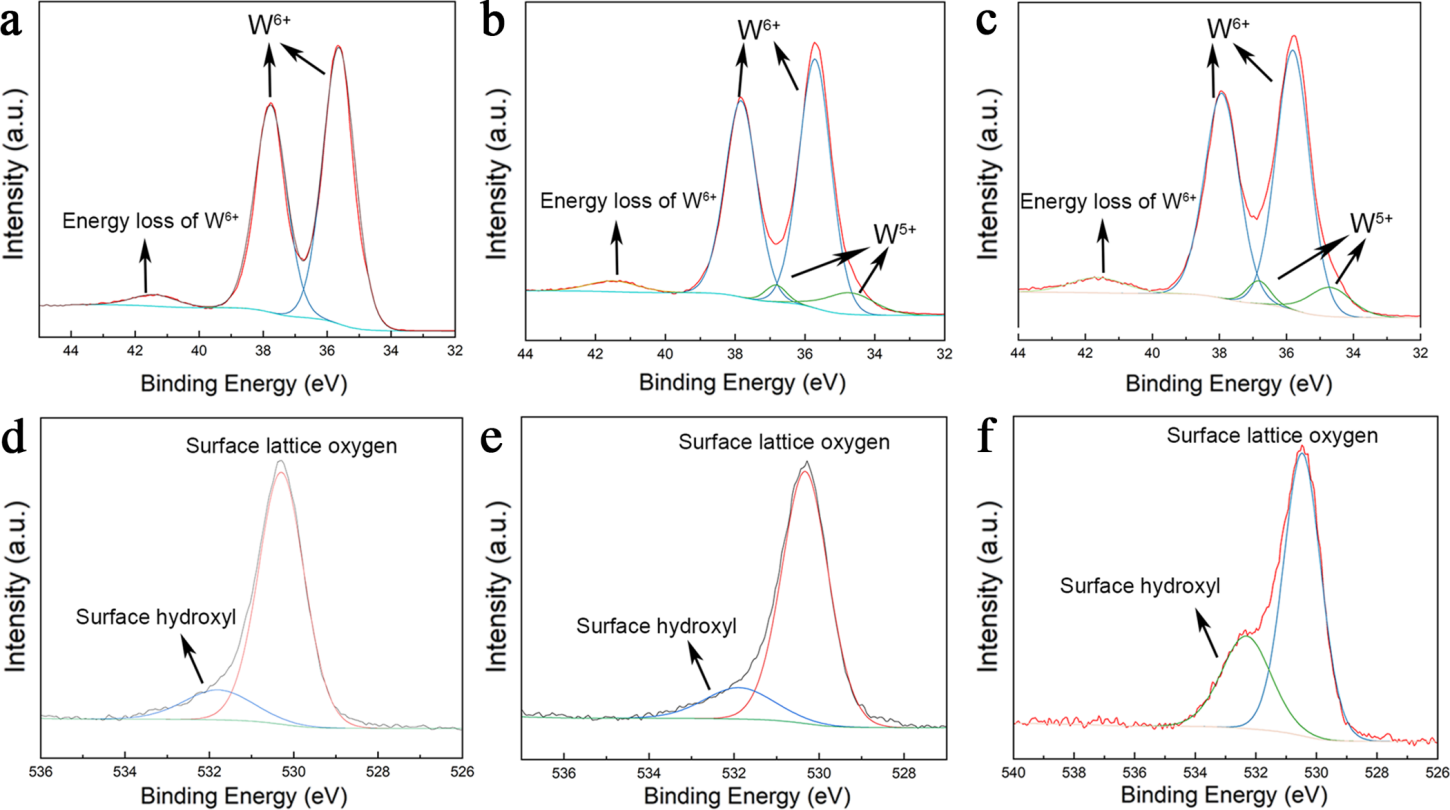
High-resolution W 4f and O 1s XPS spectra of WO_3_ nanosheets calcined under (a, d) air, (b, e) N_2_ atmosphere, and (c, f) 20% H_2_/Ar atmosphere. The increasing areas of W^5+^ and surface hydroxyl peaks indicate the generation of surface oxygen vacancies.

XPS spectroscopy provided a semiquantitative evaluation of the oxygen vacancies in the surface regions of WO_3_ nanosheets. In W 4f XPS spectra (Figures 2a–c and S3 and Table S1), the peaks of binding energies at 37.79–37.93 eV represented the W 4f_5/2_ peak of W^6+^ and the peaks at 35.65–35.81 eV corresponded to W 4f_7/2_ of W^5+^.^36^ The broad peak at around 45.1 eV corresponded to the energy loss feature of W^6+^. In XPS spectra taken from N_2_-calcined WO_3_ samples and H_2_-calcined WO_3_ samples, the additional two peaks at 36.8 and 34.7 eV, representing W 4f_5/2_ and W 4f_7/2_ of W^5+^, indicate the presence of oxygen vacancies in the surface regions. The O 1s spectra showed the peaks of surface lattice oxygen at 530.29–530.47 eV and the peaks of hydroxyl radicals at 531.79–532.31 eV on the WO_3_ surfaces.^36^ Percentage of surface hydroxyl radical sharply increased in H_2_-calcined WO_3_ comparing to samples calcined in air or N_2_. Previous work suggested that hydroxyl radical was chemisorbed to surface oxygen vacancies for stabilization.^37^ A larger area of the hydroxyl radical in the XPS spectrum indicated more oxygen vacancies on the sample surfaces. Calculated percentage of hydroxyl radicals was more than twice the ones in either air-calcined WO_3_ or N_2_-calcined WO_3_ (Table 1). It suggested that oxygen vacancies were largely generated on the surface sites. ESR (a.k.a., electron paramagnetic resonance (EPR)) spectroscopy further supported the results from XPS spectra. ESR/EPR spectroscopy showed a peak at *g* = 2.0024 where electrons were trapped at surface oxygen vacancies (Figure S4).^36,38^ These combined results suggested that WO_3_ nanosheets calcined in a 20% H_2_/Ar atmosphere were rich in surface oxygen vacancies (*V*_O_).

**Table 1:** Percentage of W^6+^, W^5+^, Surface Lattice Oxygen, and Surface Hydroxyl Radical Calculated from XPS Spectra

sample	atomic fraction %			
	W^6+^	W^5+^	surface lattice oxygen	surface hydroxyl radical
WO_3_	100	0	0.82	0.18
N_2_-calcined WO_3_	0.90	0.10	0.81	0.19
H_2_/Ar-calcined WO_3_	0.89	0.11	0.57	0.43

### Superhydrophilicity-Induced Antiprotein Adsorption Mechanism

**Figure 3:**
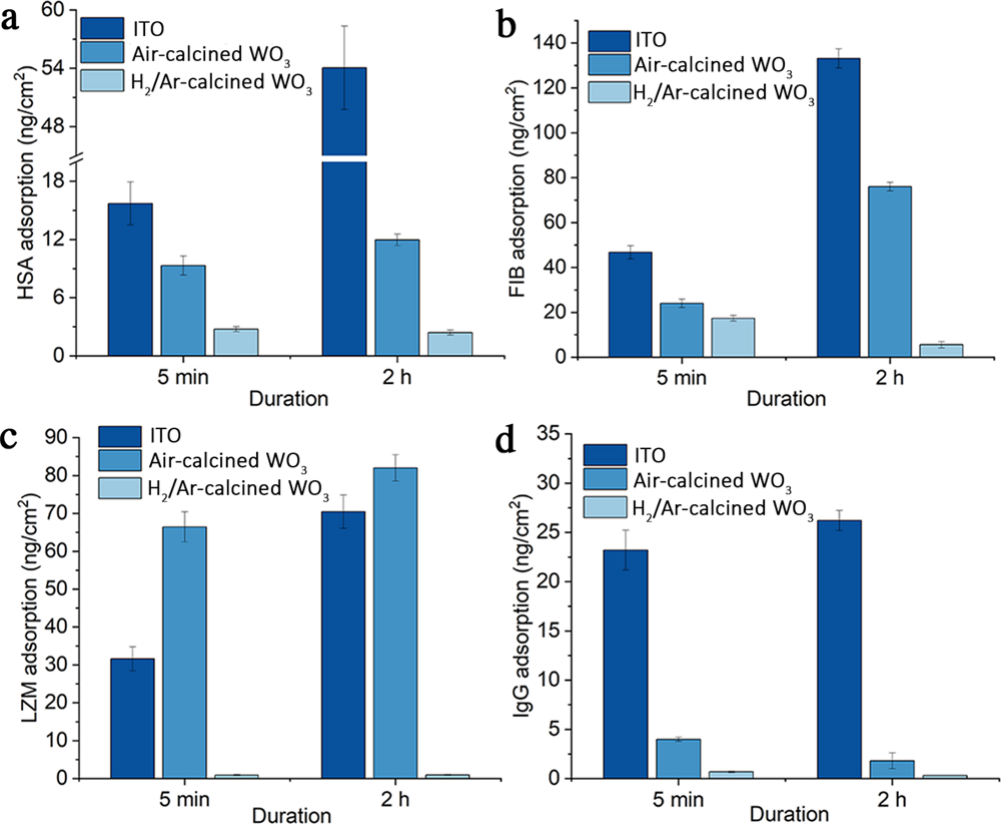
ELISA characterization of (a) HSA, (b) FIB, (c) LZM, and (d) IgG adsorbed to ITO, ITO coated with N_2_-calcined WO_3_, and ITO coated with H_2_/Ar-calcined WO_3_. Electrodes were exposed to solutions of each protein at concentrations of 1 mg/mL (*n* = 3).

**Figure 4:**
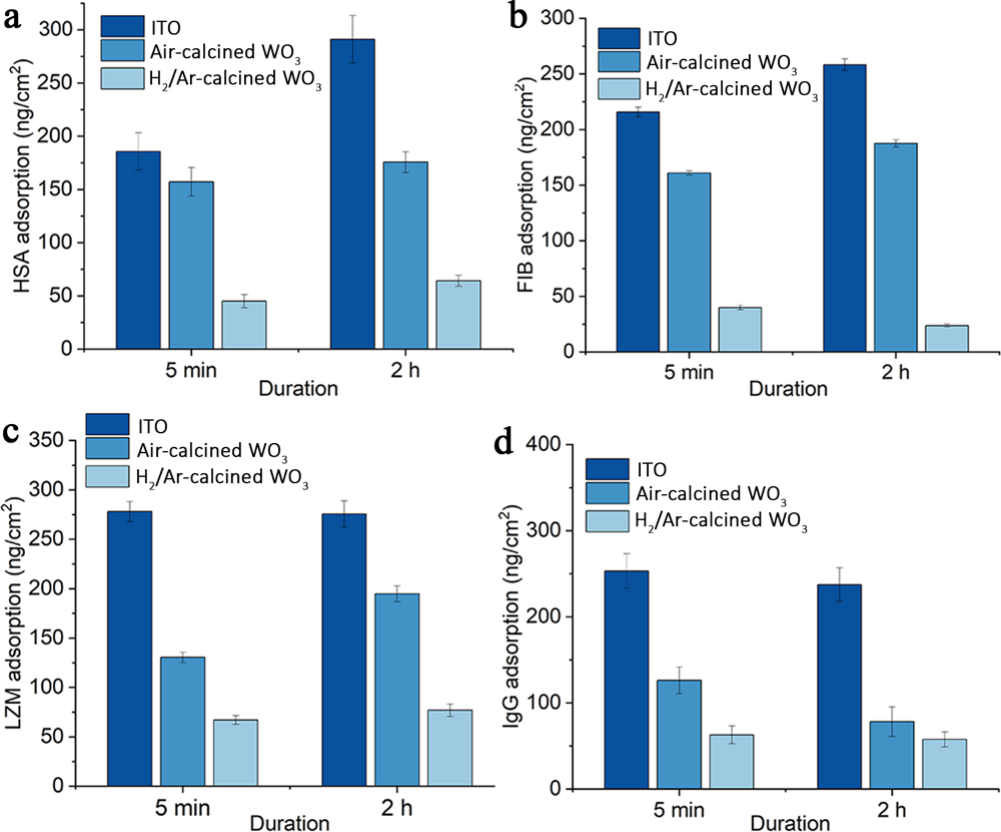
Micro-BCA characterization of (a) HSA, (b) FIB, (c) LZM, and (d) IgG adsorbed to ITO, ITO coated with N_2_-calcined WO_3_, and ITO coated with H_2_/Ar-calcined WO_3_ from unprocessed human plasma (*n* = 3).

Water contact angle is an important benchmark to characterize the wetting behavior of thin films. In our study, the commercial ITO/SiO_2_ substrate exhibited an average optical water contact angle of 36.2 ± 2.3° (Figures S5 and S6). Air-calcined WO_3_ thin films exhibited an average water contact angle of 17.5 ± 0.6°. The optical contact angles of water on N_2_-calcined WO_3_ thin films and H_2_-calcined ones were further decreased to 13.7 ± 1.1 to 8.4 ± 0.7°. To evaluate the influence of surface wetting on irreversible protein adsorption, we first incubated thin films with different water contact angles to prepare single protein solutions of HSA, FIB, LZM, and IgG for 5 min and 2 h (Figure 3). Adsorption of HSA, FIB, and LZM onto bare ITO increased significantly as the exposure time prolonged from 5 min to 2 h, while adsorption of IgG saturated at 5 min. Adsorption of HSA, FIB, and IgG to WO_3_ thin films without V_O_ was much lower than their adsorption to ITO, as optical water contact angles were decreased to 17.5 ± 0.6°. Adsorption of HSA and IgG to WO_3_ thin films saturated at 5 min, while adsorption of FIB continued to increase as incubation extended. At 5 min, adsorption of LZM to WO_3_ thin films was doubled compared to adsorption to ITO. For WO_3_ thin films rich in *V*_O_, optical water contact angles were decreased to 8.38 ± 0.7°, and adsorption of HSA, FIB, LZM, and IgG was also significantly decreased. Adsorption of HSA, LZM, and IgG remained at the same levels below 5 ng/cm^2^ as exposure extended from 5 min to 2 h, while adsorption of FIB decreased from 17.3 to 5.6 ng/cm^2^. Compared to bare ITO, adsorption of HSA, FIB, LZM, and IgG onto WO_3_ thin films rich in surface *V*_O_ was reduced by 95.5, 95.8, 98.6, and 98.7%. Compared to WO_3_ with no surface *V*_O_, the adsorption of HSA, FIB, LZM, and IgG was reduced by 79.8, 92.7, 98.8, and 82.2%, respectively. To further examine the properties of the WO_3_ films, different WO_3_ films were treated with human plasma (Figure 4). Adsorption of HSA, FIB, LZM, and IgG from human plasma was quantified using the micro-BCA method. Nonspecific protein adsorption was increased compared to prepared protein solutions due to higher protein concentrations in plasma. The trends of protein adsorption from plasma between bare ITO and ITO coated with air-calcined WO_3_ H_2_/Ar-calcined WO_3_ were the same compared with adsorption from as-prepared solutions, except for LZM. A decrease in protein adsorption at longer exposure may be attributed to the Vroman effect. Compared with bare ITO, the adsorption of HSA, FIB, LZM, and IgG onto WO_3_ thin films rich in surface *V*_O_ was reduced by 71.4, 75.2, 49.7, and 50.0%, respectively. Compared with WO_3_ with no surface *V*_O_, adsorption of HSA, FIB, LZM, and IgG was reduced by 63.4, 87.4, 60.5, and 26.3%, respectively.

### Electrochemical Biosensing with Antifouling Behavior in Plasma

**Figure 5:**
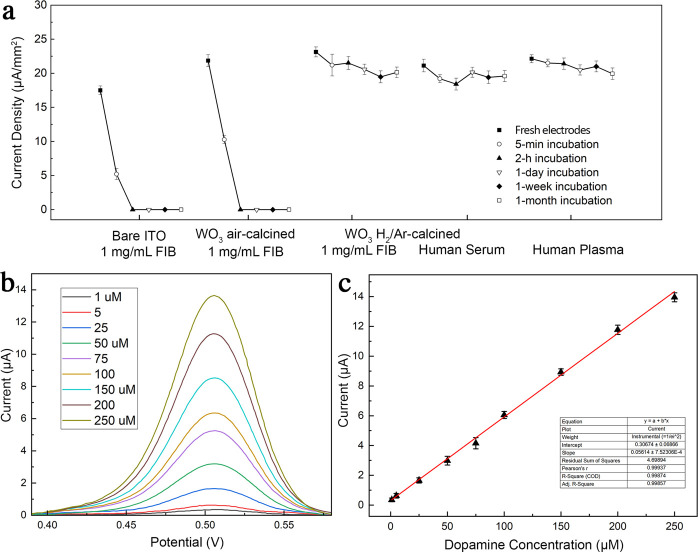
(a) Current densities measured from bare ITO electrodes, ITO coated with air-calcined WO_3_, and ITO coated with H_2_/Ar-calcined WO_3_ incubated for 1 month in 1 mg/mL FIB, human serum, or plasma. (b) SWV measurement of dopamine in human plasma using a sensor based on ITO coated with H_2_/Ar-calcined WO_3_. (c) Calibration curve of the dopamine sensor.

To demonstrate the antifouling properties of the WO_3_ thin film as the electrode surface, we incubated the sensor into FIB solution and untreated human serum and plasma (Figure 5a). After incubation for 5 min with FIB solutions, the current densities measured from bare ITO electrodes and ITO spin coated with WO_3_ thin films without *V*_O_ significantly dropped by 71 and 53%. In contrast, the current densities measured from electrodes coated with WO_3_ thin films rich in surface *V*_O_ were retained over 91%. Loss in current densities could be attributed to quick formation of FIB layers covering the electrode surfaces (Figure 3b). After 1 h of incubation in FIB solution, current densities from bare ITO and ITO coated with WO_3_ with no *V*_O_ completed decreased to zero, since the layers of absorbed FIB completed hindered charge transfer between the electrodes and redox species in electrolytes. On the other hand, WO_3_ rich in *V*_O_ maintained around 92% of their maximum current densities. Their current densities even remained for more than 87% even though the electrodes had been incubated for one month in either undiluted human serum or plasma. We further demonstrated electrochemical biosensor based on WO_3_ rich in *V*_O_ to detect dopamine in human plasma with antifouling performances. The electrochemical detection of dopamine was based on SWV at a fixed scan rate of 50 mV/s (Figure 5b). The oxidation peak of dopamine was measured at around 0.503 V. The clear peaks indicated effective electrocatalyzation of dopamine in plasma without biofouling from nonspecific adsorption of proteins. The oxidation peak increased monotonically as the concentration of dopamine increased. The dopamine sensor exhibited a linear range of detection from 1 to 250 μM without biofouling from plasma (Figure 5c).

### Nanotoxicity of WO_3_ Nanosheets with Surface Oxygen Vacancies

**Figure 6:**
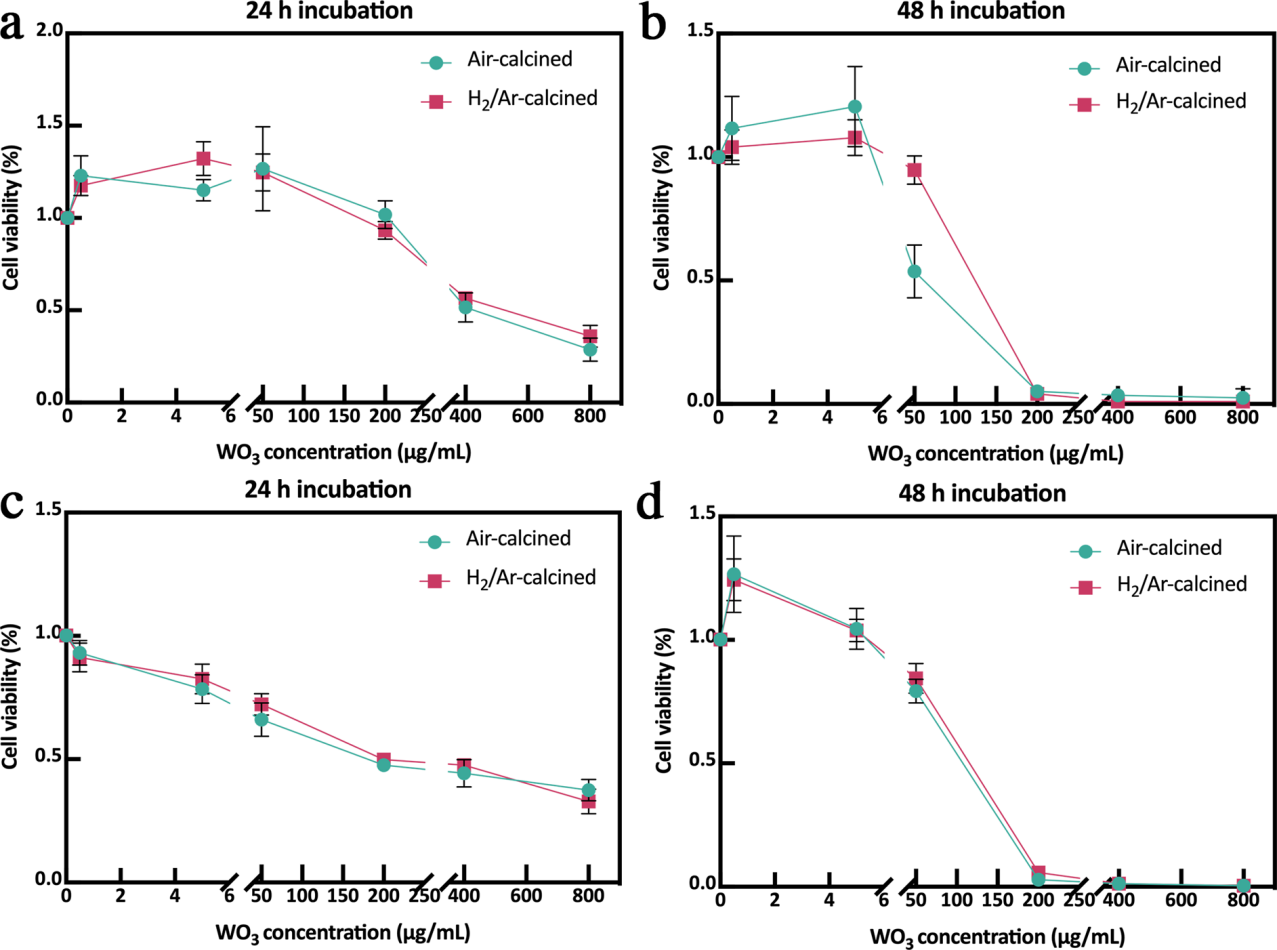
Cell viability of HUVECs after incubation with WO_3_ calcined in air or 20% H_2_/Ar, evaluated by (a, b) CCK-8 assay and (c, d) CTG assay (*n* = 3). Cell viability was calculated in percentage of the control group.

Effects of tungsten trioxide on human somatic cells remain mostly unknown. When applied as electrodes of implanted sensors, WO_3_ nanosheets potentially falling off from the thin film might cause potential damage to blood veins. Therefore, we selected HUVECs to test the toxicity of the WO_3_ nanosheets. We incubated HUVECs with concentrations (0–800 μg/mL) of WO_3_ nanosheets which were rich in surface oxygen vacancies for 24 and 48 h. Cell cytotoxicity was evaluated by both the CCK-8 and CTG assays. From CCK-8 results (Figure 6a,b), WO_3_ with and without *V*_O_ both slightly promoted cell growth after 24 and 48 h incubation when concentrations of WO_3_ nanosheets were between 0.5 to 5 μg/mL. When concentration further increased to 50 ng/mL, activity of HUVECs started to decrease. At a concentration of 200 ng/mL, HUVECs exhibited no activity after 48 h of incubation. It suggested WO_3_ exhibited a two-sided effect on HUVECs which was both dose- and time-dependent, which could be attributed to DNA damage caused by WO_3_ uptake (Figure S7).

## Conclusions

We reported an antibiofouling strategy by controlling oxygen vacancies in WO_3_. By increasing surface oxygen vacancies, we improved the water contact angles of WO_3_ thin films from 34 to 8°. As a result of superhydrophilicity, biofouling on the WO_3_/ITO electrodes was significantly reduced by more than 95% compared to bare ITO electrodes after exposure to model protein solutions at 1 mg/mL. Protein adsorption from serum was reduced by 76%. After exposure to human serum and plasma for 1 month, current densities measured from electrodes based on WO_3_ rich in surface oxygen vacancies maintained over 91%. When the concentration was as low as 500 ng/mL in the medium, WO_3_ rich in oxygen vacancies also exhibited low toxicity to HUVECs. We believed that the antibiofouling behavior arose from introduction of oxygen vacancy sites in cubic WO_3_ nanosheets. Oxygen vacancies bound to hydroxyl radicals, which led to superhydrophilicity and energy-favored formation of a water layer on the surface of WO_3_ thin films. The tightly bound water layer served as an obstacle to the nonspecific proteins in human serum.

## Data Availability

The data sets generated during and/or analyzed during the current study are available from the corresponding author on reasonable request.

## Supporting Information Available

Additional experimental details including dynamic light scattering data for the size distribution of WO_3_ nanosheets, Raman spectrum, XPS spectra, ESR/EPR spectra for WO_3_, water contact angle measurement of thin films, and cell cytotoxicity characterized from caspase-3 and 8-oxo-dG (PDF)
