# Supplementary material for: Surface Oxygen Deficiency Enabled Spontaneous Antiprotein Fouling in WO3 Nanosheets for Biosensing in Biological Fluids
Source: Anal Chem. 2024 Jan 4;96(2):839–46. doi: 10.1021/acs.analchem.3c04414 (PMC10794997; doi:10.1021/acs.analchem.3c04414)
Supplement: Supplementary file 1 — ac3c04414_si_001.pdf [file ac3c04414_si_001.pdf]

Supporting Information

Surface Oxygen Deficiency Enabled Spontaneous Anti-protein-fouling in WO_3 Nanosheets for Biosensing in Biological Fluids

Guozhen He[a,b,d], Tao Dong[b,*], Zhaochu Yang[a,d,*], Bjørn Torger Stokke[b,c], Zhuangde Jiang[a,e]

(The following affiliations are listed in no particular order and are listed in the alphabetical order by name abbreviation:

[a] Chongqing Key Laboratory of Micro-Nano Systems and Smart Transduction, Chongqing Key Laboratory of Colleges and Universities on Micro-Nano Systems Technology and Smart Transducing, Collaborative Innovation Center on Micro-Nano Transduction and Intelligent Eco-Internet of Things, Chongqing Academician and Expert Workstation, Chongqing Technology and Business University, Nan'an District, 400067 Chongqing, China

[b] Department of Microsystems (IMS), Faculty of Technology, Natural Sciences and Maritime Sciences, University of South-Eastern Norway, Postboks 235, 3603 Kongsberg, Norway

[c] Biophysics and Medical Technology, Department of Physics, Norwegian University of Science and Technology, NO-7491 Trondheim, Norway

[d] Sensovann AS, Raveien 215, 3184 Borre, Norway.

[e] Xian Jiaotong University, 710049 Xian, China

*Corresponding authors: tao.dong@usn.no; Zhaochu.yang@ctbu.edu.cn

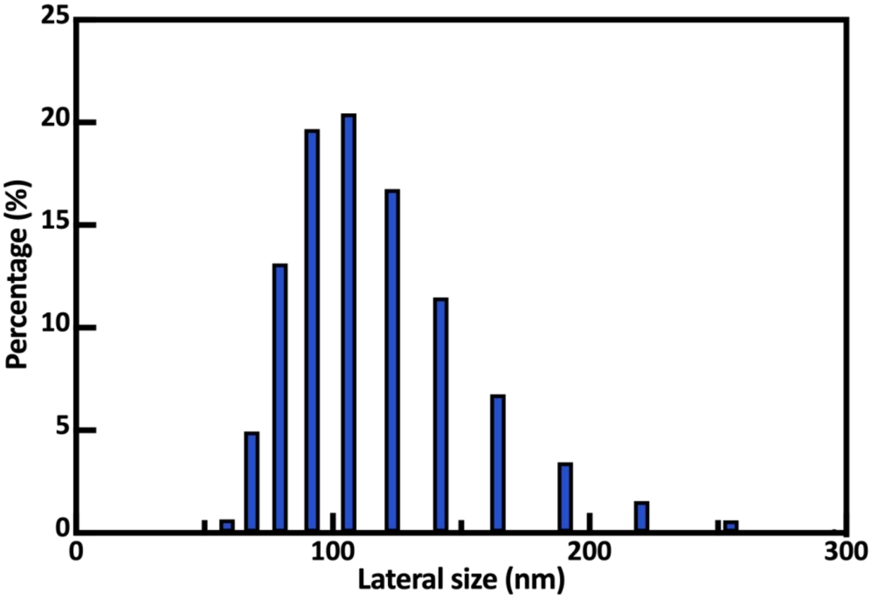

Figure S1. Size distribution of as-synthesized WO_3 clusters without probe sonication measured by dynamic light scattering. The average lateral size was 138.7 nm.

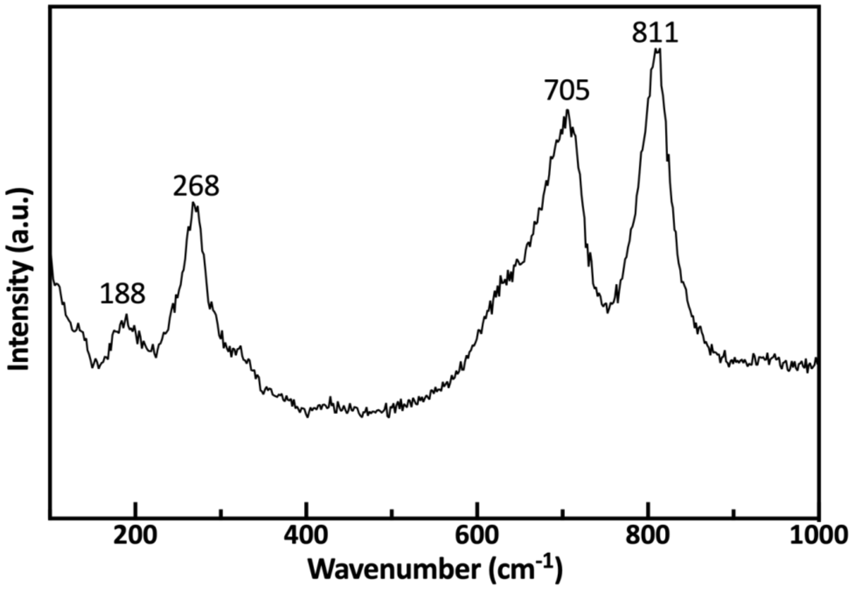

Figure S2. Raman spectrum of WO_3 nanosheets calcined in 20% H_2/Ar atmosphere.

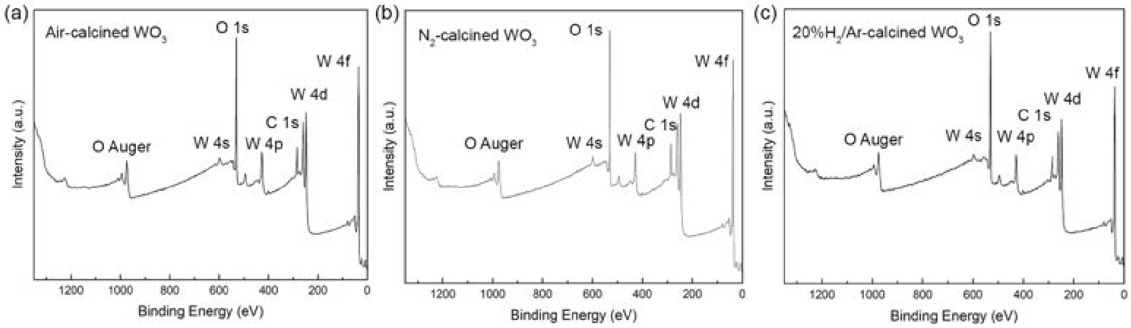

Figure S3. XPS spectra of WO_3 calcined under (a) air, (b) N_2 atmosphere, and (c) 20% H_2/Ar atmosphere.

Table S1. Binding energy of tungsten and oxygen peaks summarized from XPS spectra.

Sample	Peak binding energy (eV)					
	W^{6+}		W^{5+}			Surface hydroxyl
	$4f_{7/2}$	$4f_{5/2}$	$4f_{7/2}$	$4f_{5/2}$	Surface oxygen	radical
WO_3	35.65	37.79			530.29	531.79
N_2-calcined WO_3	35.71	37.83	34.71	36.83	530.33	531.88
H_2/Ar-calcined WO_3	35.81	37.93	34.71	36.83	530.47	532.31

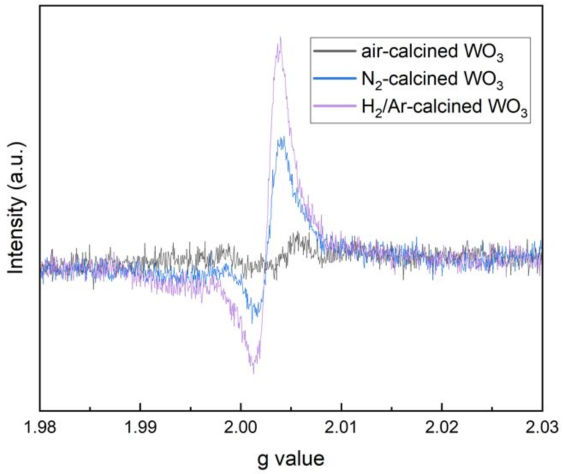

Figure S4. ESR/EPR spectra of WO_3, peak at g=2.0024.

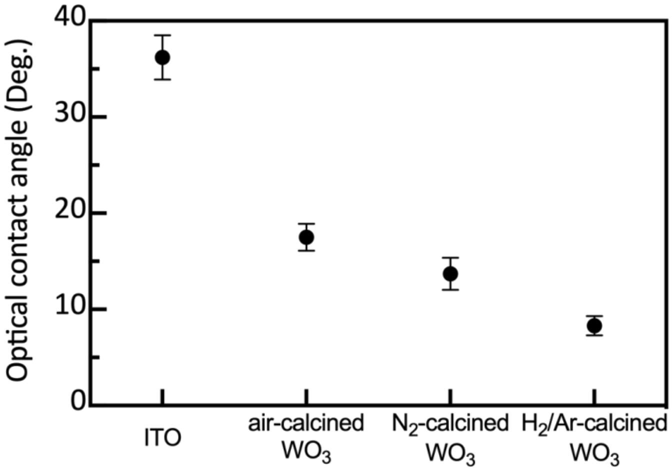

Figure S5. Optical contact angles of water measured on ITO substrates and ITO coated with WO_3 calcined in different environment (n=4).

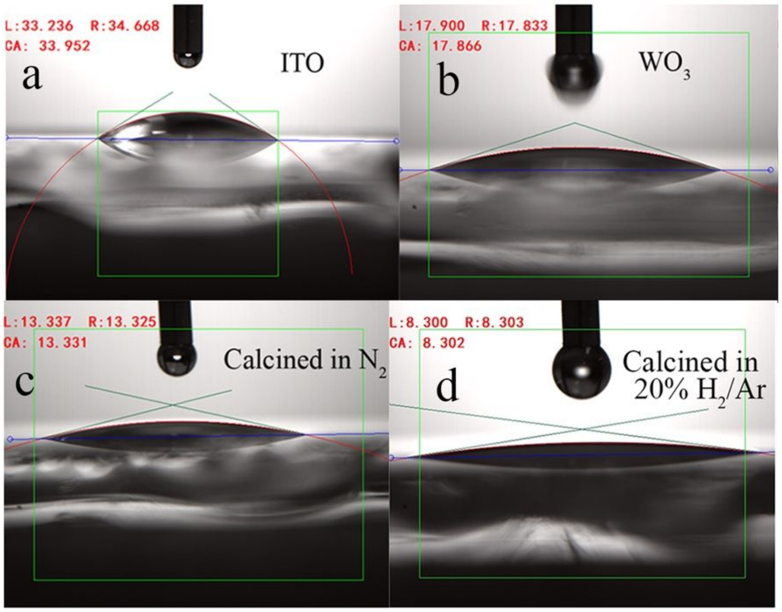

Figure S6. Pictures of contact angles of water on (a) ITO substrate, (b) ITO coated with air-calcined WO_3, (c) ITO coated with N_2-calcined WO_3, and (d) ITO coated with H_2/Ar-calcined WO_3 thin films.

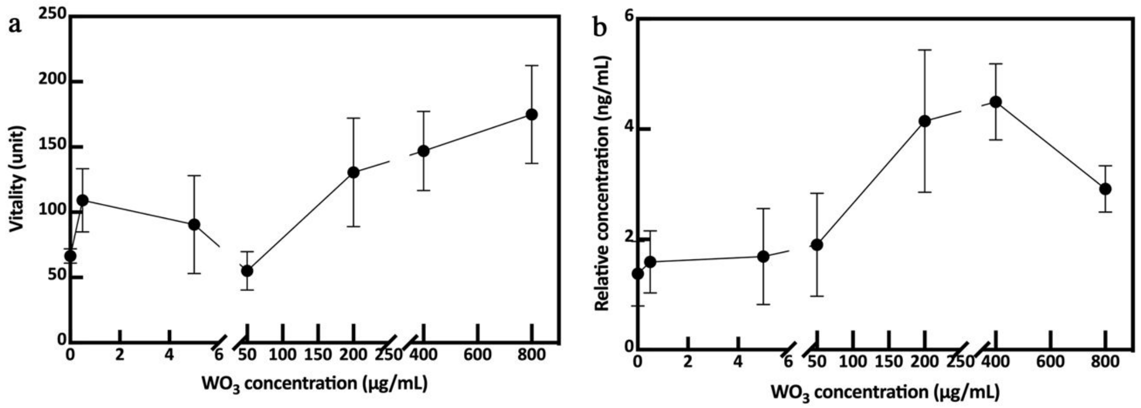

Figure S7. Apoptosis and DNA damage of HUVECs by WO_3 nanosheets, evaluated by (a) units of caspase-3 per unit weight and (b) 8-oxo-dG concentrations in cell supernatant. (n=3)
